# Dysferlinopathy Fibroblasts Are Defective in Plasma Membrane Repair

**DOI:** 10.1371/currents.md.5865add2d766f39a0e0411d38a7ba09c

**Published:** 2015-10-29

**Authors:** Chie Matsuda, Kazuyuki Kiyosue, Ichizo Nishino, Yuichi Goto, Yukiko K. Hayashi

**Affiliations:** Biomedical Research Institute, National Institute of Advanced Industrial Science and Technology (AIST), Central 6, 1-1-1 Higashi, Tsukuba, Ibaraki 305-8566, Japan; Department of Neurophysiology, Tokyo Medical University, Shinjuku, Tokyo 160-8402, Japan; Department of Neuromuscular Research, National Institute of Neuroscience, National Center of Neurology and Psychiatry (NCNP), 4-1-1 Ogawa-Higashi, Kodaira, Tokyo 187-8502, Japan; Biomedical Research Institute, AIST, 1-8-31 Midorigaoka, Ikeda 563-8577, Japan; Department of Neuromuscular Research, National Institute of Neuroscience, National Center of Neurology and Psychiatry (NCNP), 4-1-1 Ogawa-Higashi, Kodaira, Tokyo 187-8502, Japan; Department of Genome Medicine Development, Medical Genome Center (MGC), National Institute of Neuroscience, NCNP, 4-1-1 Ogawa-Higashi, Kodaira, Tokyo 187-8502, Japan; Department of Genome Medicine Development, Medical Genome Center (MGC), National Institute of Neuroscience, NCNP, 4-1-1 Ogawa-Higashi, Kodaira, Tokyo 187-8502, Japan; Department of Mental Retardation and Birth Defect Research, National Institute of Neuroscience, NCNP, 4-1-1 Ogawa-Higashi, Kodaira, Tokyo 187-8502, Japan; Department of Neurophysiology, Tokyo Medical University, Shinjuku, Tokyo 160-8402, Japan; Department of Neuromuscular Research, National Institute of Neuroscience, National Center of Neurology and Psychiatry (NCNP), 4-1-1 Ogawa-Higashi, Kodaira, Tokyo 187-8502, Japan

## Abstract

Background: Dysferlin is a sarcolemmal protein that is defective in Miyoshi myopathy and limb-girdle muscular dystrophy type 2B, and is involved in sarcolemmal repair. Primary cultured myoblasts and myotubes established from patient muscle biopsies have been widely utilized to explore the molecular mechanism of dysferlinopathy.

Objectives: The purpose of this study was to explore the possible utility of dermal fibroblasts from dysferlin-deficient patients and SJL mice as a tool for studying dysferlinopathy.

Methods: Dysferlin protein expression in fibroblasts from dysferlin-deficient patients and SJL mice was analyzed by immunoblotting and immunocytochemistry. The membrane wound-repair assay was performed on the fibroblasts using a confocal microscope equipped with a UV-laser. The membrane blebbing assay using hypotonic shock, in which normal membrane blebbing is detected only in the presence of dysferlin, was also performed using human and mouse fibroblasts.

Results: Mis-sense mutated dysferlin was expressed at a very low level in fibroblasts from a dysferlinopathy patient, and lower expression level of truncated dysferlin was observed in SJL mouse fibroblast. Fibroblasts from patients with dysferlinopathy and SJL mice showed attenuated membrane repair and did not form membrane blebs in response to hypoosmotic shock. Proteosomal inhibitior increased mis-sense mutated or truncated dysferlin levels, and restored membrane blebbing, however, proteosomal inhibition failed to improve levels of dysferlin with non-sense or frame-shift mutation.

Conclusion: Fibroblasts from dysferlinopathy patients and SJL mice showed attenuated plasma membrane repair, and could be a tool for studying dysferlinopathy.

## Introduction

Mutations in the dysferlin gene (*DYSF*) cause myopathies including Miyoshi myopathy (MM), limb girdle muscular dystrophy type 2B (LGMD2B), and distal anterior compartment myopathy^1^^,^ 2 ^,^ 3, which are collectively called the dysferlinopathies. From the results that dysferlin accumulates at sarcolemmal injury sites in a Ca^2+^-dependent manner, dysferlin is thought to be involved in membrane resealing 4, and insufficient sarcolemmal repair caused by dysferlin deficiency is thought to be closely related to the muscle degeneration in dysferlinopathy. To explore the molecular mechanism of dysferlinopathy, previous studies utilized primary cultured myoblasts and myotubes established from human muscle biopsies 5 ^,^ 6.

Here, we show insufficient plasma membrane repair in fibroblasts from dysferlinopathy patients and SJL mice expressing truncated dysferlin. We also demonstrate that mutant fibroblasts do not form membrane blebs in response to hypotonic shock, which is clearly observed in normal fibroblasts. Sinnreich *et al.* reported that the proteasome inhibitor increased mis-sense mutated dysferlin in human myoblast cultures 6. We found that proteasome-specific inhibitor MG-132 can increase the levels of the truncated dysferlin protein in SJL fibroblasts and mis-sense mutated dysferlin in the patient fibroblasts. In addition, the salvaged mutant dysferlin can efficiently restore membrane blebbing. Our results suggest the possibility that fibroblasts can be used as a research tool for dysferlinopathy, and that proteasome inhibitor may be a potential treatment for dysferlinopathy patients harboring truncated or mis-sense mutated dysferlin.

## Materials and Methods

**Ethics Statement**

All experiments involving human specimens were approved by the Research Ethics Committees of the National Institute of Neurology and Psychiatry (NCNP) and the National Institute of Advanced Industrial Science and Technology (AIST), and were performed in accordance with their guidelines. All experiments involving animals were approved by the Institutional Animal Care and Use Committee of AIST and performed according to the Procedure for Handling Experiments involving Animals of AIST.

**Primary fibroblast cultures**

We obtained three human primary fibroblasts from NCNP BioBank, along with the required IRB approval. Control fibroblast has no mutation in *DYSF*. Mutations in *DYSF* of two patient fibroblast cultures were summalized in Tabale 1. For isolation of mouse fibroblasts, SJL mice on a C57BL/6J background (SJL/B6) that were obtained by crossing SJL/J and C57BL/6J mice for more than 10 generations were used. Genotyping of the SJL/6J mice was performed as previously described 7. Skins from SJL/6J and C57BL/6J mice were minced and then trypsinized in 0.25% trypsin (Sigma) containing 0.4% collagenase type II (Gibco). The trypsinized skins were pelleted down by centrifugation and resuspended in Dulbecco’s modified Eagle’s medium MEM (DMEM; Sigma) containing 10% fetal bovine serum (FBS; Gibco) 8. All fibroblast were cultured in DMEM containing 10% FBS at 37 ℃ and 5% CO_2_ in a humidified incubator. Mouse fibroblasts up to passage 3, which grow rapidly, were used in the experiments.

**Immunoblotting**

Cells were lysed in sample buffer containing 62.5 mM Tris-HCl/pH 6.8, 2% SDS, 10% glycerol, 5% 2-mercaptoethanol and 0.002% bromophenol blue, and sonicated briefly on ice. Lysates were separated by electrophoresis on 5-20% SDS-polyacrylamide gels and transferred onto nitrocellulose membranes (Hybond ECL, GE Healthcare). Membranes were subjected to immunoblotting with the anti-dysferlin monoclonal antibody (NCL-Hamlet and NCL-Hamlet-2, Leica, 1:1000) and anti-GAPDH rabbit polyclonal antibody (G9545, Sigma, 1:1000), followed by incubation with ECL (GE Healthcare). Detection was performed using the ChemiDoc^TM^ XSR system (Bio-Rad). Densitometric analysis was performed using FIJI (http://fiji.sc/Fiji).

**Immunocytochemistry**

Fibroblasts were fixed in 2% paraformaldehyde for 10 minutes and permeabilized in 0.1% Triton X-100 for 5 minutes at room temperature. Nonspecific immunoreactions were blocked by a 1 hour incubation with 5% goat serum and 2% bovine serum albumin in PBS. Cells were incubated with the anti-dysferlin mouse monoclonal antibody (NCL-Hamlet, 1:300) for 2 hours at 37 ℃, followed by incubation with Cy3-labeled anti-mouse IgG (Jackson Laboratories, 1:500) for 1 hour.

**Membrane repair assay**

Human and mouse fibroblasts were plated onto glass-bottom dishes (Matsunami Glass). Before laser wounding, cells were rinsed with Tyrode solution containing 10 mM HEPES, 140 mM NaCl, 5 mM KCl, 2.5 mM CaCl_2_ and 2 mM MgCl_2_, and then incubated in the same solution containing 2.5 mM FM1-43 (Molecular Probes). Fibroblasts were wounded by irradiating a 10 mm (human) or 6 mm (mouse) diameter circular area of the plasma membrane at 100% maximum power for 20 sec (405-nm UV laser) using the laser confocal microscope FLUOVIEW FV1000MPE (Olympus). Images were captured at 5-second intervals. Resealing analysis based on the kinetics and extent of FM1-43 entry through open disruptions was carried out as previously described 4.

**Hypotonic shock**

Human and mouse fibroblasts were plated onto glass-bottom dishes. Before hypotonic osmotic shock, cells were rinsed with Tyrode solution. For creating hypotonic osmotic shock 5, cells were exposed to a hypotonic solution (50% dilution of Tyrode solution by water) containing 2.5 mM FM1-43. Membrane blebbing was observed with a laser confocal microscope.

**Treatment with the proteasome inhibitor MG-132**

Human and mouse fibroblasts were seeded onto 24-well plates and glass-bottom dishes. Semi-confluent cells were treated with three concentrations (10 nM, 100 nM and 1 µM) of MG-132 (Peptide Institute) for 24 hours in a CO_2_ incubator. Cells were subjected to the quantification of dysferlin protein by immunoblotting or membrane blebbing assay.

## Results

**Dysferlin protein expression in fibroblasts.**

Northern blot analyses previously revealed that dysferlin transcripts are ubiquitously expressed 1, however, it remains unclear whether the dysferlin protein is expressed in fibroblasts. To investigate dysferlin protein expression in human and mouse skin fibroblasts, lysates were prepared from cultured fibroblasts and subjected to SDS-PAGE followed by immunoblotting. The dysferlin antibody (NCL-Hamlet) used for immunoprobing has been reported to react with both human and mouse dysferlin 9. Dysferlin was detected in skin fibroblasts from both normal humans and wild-type mice, whereas it was barely detectable in fibroblasts from dysferlinopathy patients (patient 1 and 2, Fig. 1). In accordance with a previous report 10, SJL/6J mouse fibroblasts expressed a truncated 229-kDa dysferlin protein at lower levels compared with full-length dysferlin in the fibroblasts of wild-type mice (Fig. 1).

**Table:** **Table 1: DYSF mutations in the human fibroblasts**

Fibroblast culture	Mutation	Predicted protein	Predicted MW
Normal control	No mutation	No mutaion	237 kDa
Patient 1	c.2997 G>T homozygote	W999C	237 kDa
Patient 2	c.1566C>G c.5698_5699delAG	Y522X S1900Qfs	57.7 kDa 217 kDa

**Immunoblot analysis of human and mouse fibroblasts using an anti-dysferlin antibody:**
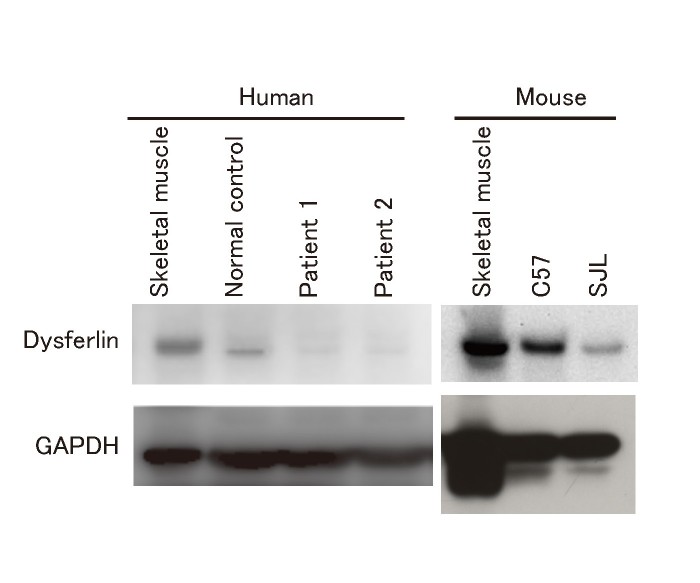
Normal 237-kDa dysferlin was detected in control human and wild-type mouse fibroblasts. Dysferlin was expressed at a very low level in fibroblasts from a dysferlinopathy patient, whereas a smaller 229-kDa dysferlin was detected in SJL/6J mice.

**Subcellular localization of dysferlin in fibroblasts**

To examine the subcellular localization of dysferlin, fibroblasts were immunolabeled using anti-dysferlin antibody. Dysferlin was observed as cytoplasmic vesicles in fibroblasts from normal humans and both wild-type and SJL/6J mice (Fig. 2). Unlike human myotubes 11, wild-type dysferlin was not directed to plasma membrane in human and mouse fibroblasts. In accordance with the results of immunoblotting (Fig. 1), the immunoreaction to dysferlin was extremely low in the patient fibroblasts (Fig. 2).

**Immunostaining of human and mouse fibroblasts using anti-dysferlin:**
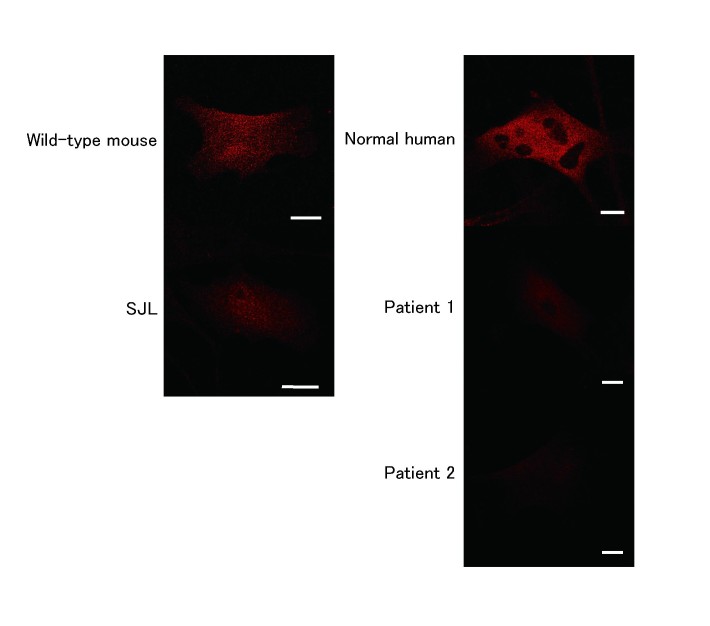
Cytoplasmic staining of dysferlin is seen in fibroblasts from control humans, wild-type mice, and SJL/6J mice, whereas dysferlin staining was barely detectable in fibroblasts from the patients. Scale bars, 20 µm.

**Membrane repair in fibroblasts**

To examine the membrane repairing capabilities of the fibroblasts, we performed the membrane repair assay using a UV-laser. Normal human and mouse fibroblasts show focal and limited accumulation of FM1-43 after UV-laser irradiation, indicating complete membrane repair (Fig. 3 and 4). By contrast, fibroblasts from patients and SJL/6J mice showed continuous entry of FM1-43 into the cells, indicating attenuated membrane repair (Fig. 3　and 4). These results indicate that mutations in the dysferlin gene lead to defective membrane repair in fibroblasts, which is similar to what is observed in myofibers.

**Membrane repair assay of human and mouse fibroblasts:**
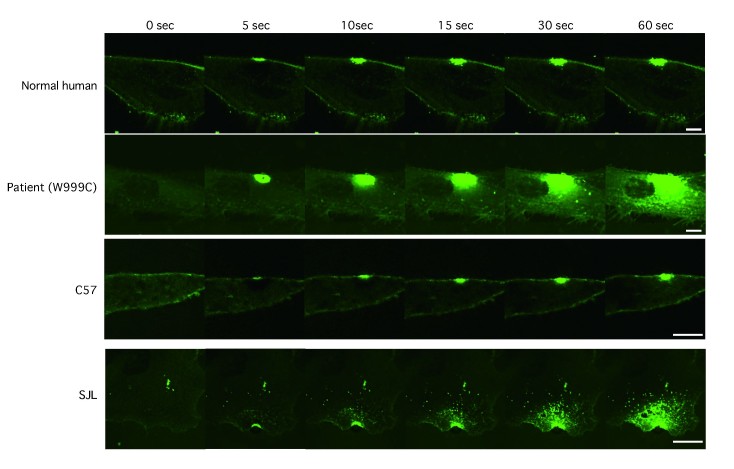
The panel shows entry of the FM 1-43 dye after laser wounding in human and mouse fibroblasts. Scale bars, 20 µm.

**Quantification of FM1-43 dye influx after laser injury:**
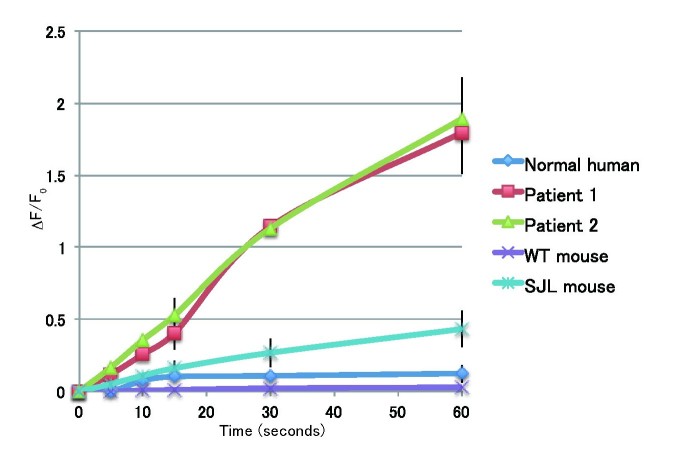
Time course relative fluorescence intensity of FM1-43 at wounded site. Data are presented as means ± S.D.(error bars). Number of laser-induced injuries; n=5.

**Dysferlin-dependent membrane blebbing in fibroblasts**

Sweeney *et al.* reported that membrane blebbing that occurs in response to hypotonic shock requires the presence of dysferlin in human and mouse cultured myotubes 5. These results suggest that fibroblasts expressing dysferlin might also form membrane blebs. To test this possibility, we performed the membrane blebbing assay using human and mouse fibroblasts. Fibroblasts from normal humans and mice show membrane blebs after 10-20 seconds of exposure to hypotonic shock (Fig. 5), similarly to myotubes 5. In contrast, no blebs were observed in the fibroblasts from the dysferlinopathy patients, and few blebs were observed in SJL/6J mice (Fig. 5 and 6). These results demonstrate that mutations in the dysferlin gene lead to defective membrane blebbing in response to hypotonic shock in fibroblasts, which is similar to what is observed in myotubes.

**Membrane blebbing assay of human and mouse fibroblasts:**
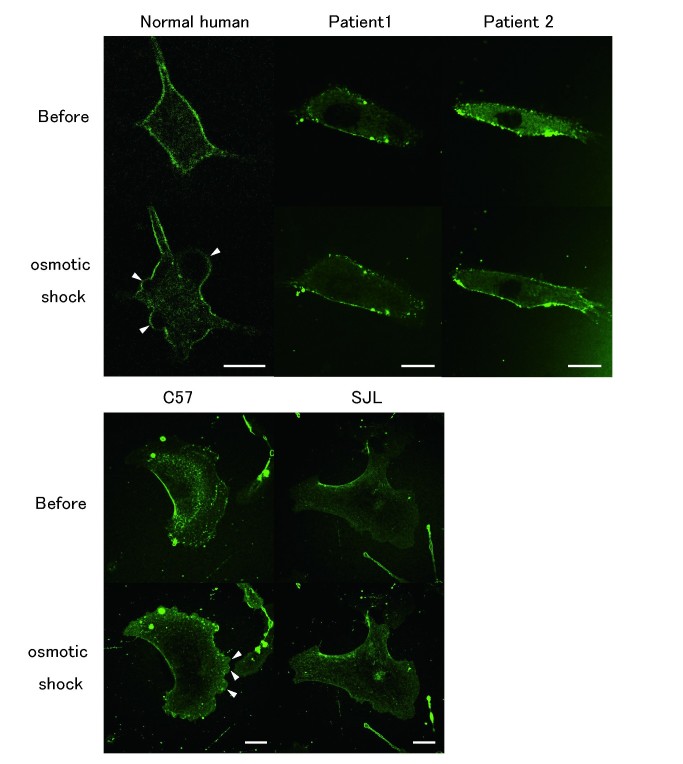
Hypotonic shock using Tyrode buffer diluted by 50% with water was performed in the presence of FM1-43. Membrane blebs are not observed in the mutant fibroblasts from a patient. Both wild-type and mutant fibroblasts with endocytic uptake of FM dye showed increased fluorescence intensity in the intracellular space. Scale bars, 20 μm.

**Quantification of membrane bleb formation in response to hypotonic shock:**
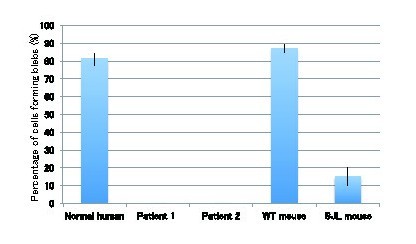
Bar graph depicting the percentage of cells manifesting blebs after osmotic shock (n=30). Data are presents as means ±s.d.

**A proteasomal inhibitor increases protein levels of truncated or mis-sense mutated dysferlin**

Sinnreich *et al.* reported that proteasomal inhibitors such as bortezomib and lactacystin improve the expression level of R555W mutant dysferlin in cultured human myoblasts 6. However, it remains unclear whether proteasomal inhibitors are effective in improving the expression level of truncated or other mis-sense mutated dysferlin in fibroblasts. Hence, we treated fibroblasts with the proteasomal inhibitor MG-132, and quantified the protein levels of dysferlin by immunoblot analysis. NCL-Hamlet, the antibody that we used for immunoblotting recognizes an epitope at the C-terminus of human dysferlin (amino acids (aa) 1999-2016, corresponding to aa 1988-2005 of mouse dysferlin), and can detect truncated dysferlin lacking the C2E domain (aa 1603-1689) expressed in SJL/6J mice 10. We also used NCL-Hamlet-2 that recognizes amino acids 349-366 of human dysferlin to detect mutant dysferlin in patient 2. Inhibition of the proteasome pathway using MG-132 lead to elevated protein levels of wild- type, truncated and mis-sense mutated dysferlin in the fibroblasts, but not in the fibroblast from patient 2 with non-sense and frame shift mutations (Fig. 7 and 8). Our results demonstrate that normal, truncated and mis-sense mutated dysferlin are degraded by the proteasome. No notable effects on viability were detected as a result of the increased levels of dysferlin in the wild-type fibroblasts.

**Immunoblot analysis of human and mouse fibroblasts after the treatment with proteosomal inhibitor:**
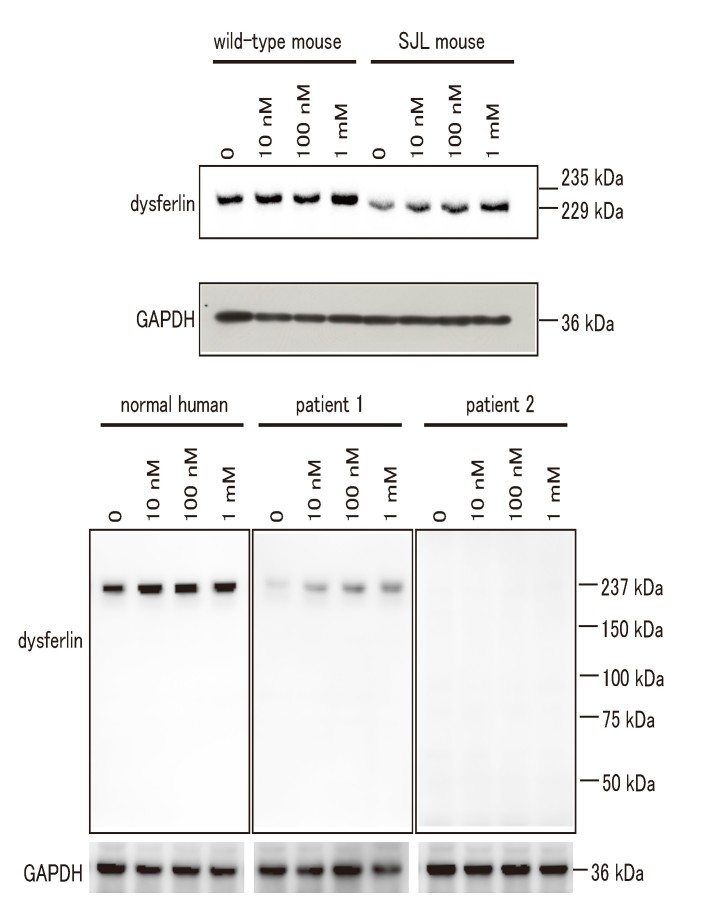
Semiconfluent culture of human and mouse fibroblasts were treated with increasing concentration of the proteasomal inhibitor MG-132. Anti-dysferlin and anti-GAPDH antibodies were used for immunoblotting.

**Relative dysferlin levels upon MG-132 treatment:**
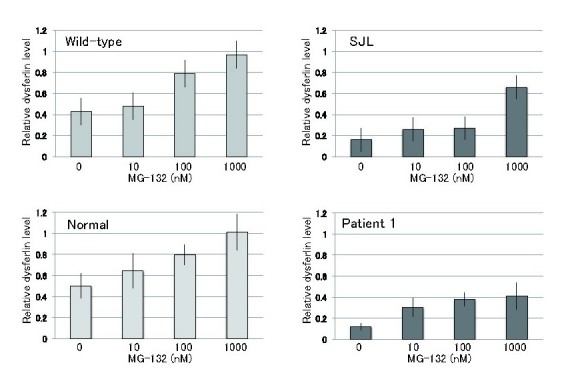
Relative dysferlin protein expression levels upon MG-132 treatment. Protein levels were measured by densitometric analysis. GAPDH was used as a loading control, and y axis shows the ratio between dysferlin and GAPDH (n =5). Data are presents as the means ± s.d.

**Truncated or mis-sense mutated dysferlin can restore membrane blebbing**

To examine whether the mutant dysferlin salvaged by MG-132 is biologically functional, we performed blebbing assay using MG-132-treated fibroblasts. MG-132-treated fibroblasts from patient 1 and SJL mice showed recovery of membrane blebbing in a dose-dependent manner (Fig. 9). In contrast, MG-132-treated fibroblast from patient 2 did not form membrane blebbing inresponse to hypotonic shock (data not shown).

**Membrane blebbing assay using MG-132-treated fibroblasts from the patients and SJL mice:**
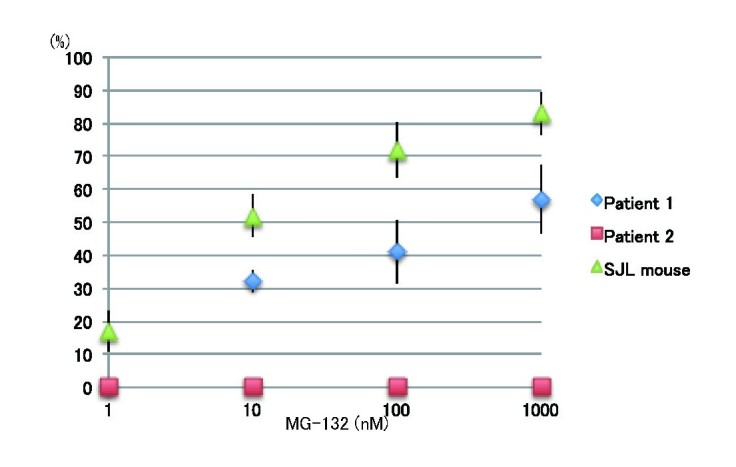
Bar graph depicting the percentage of cells manifesting blebs after osmotic shock (n=30). Data are presents as means ±s.d. Mis-sense mutated dysferlin recovered membrane blebbing as similar to truncated dysferlin in SJL mice.

## Discussion

Mutations in *DYSF* cause progressive muscular dystrophies that present clinical symptoms exclusively in skeletal muscles. For this reason, myoblasts and myotubes are used for studying dysferlinopathy. Previously, McNally *et al.* reported that dysferlin-null fibroblasts accumulated transferrin and acidic vesicles labeled with LAMP-2 similar to myoblast, however, they did not examine the capability of membrane repair in dysferlin-null fibroblasts 12. Here, we demonstrate that normal fibroblasts express the dysferlin protein, whereas those from a dysferlinopathy patient and SJL/6J mice show decreased dysferlin protein levels as well as insufficient membrane repair, similar to that observed in dysferlin-deficient myotubes. This is the first report about attenuated membrane repair in dysferlinopathy fibroblast. There are many reports about attenuated membrane repair in dysferlinopathy skeletal muscle 4 ^,^ 13 and heart 14, whereas, the ability of membrane repair in dysferlinopathyt fibroblasts have not been explored.

Skin biopsies are performed for diagnosis of merosin negative congenital muscular dystrophy 15, Ullrich congenital muscular dystrophy 16 and dystrophinopathy 17. Fibroblasts established from skin biopsy were myogenically converted by MyoD, and were used for study of the dysferlinopathy treatment 18 ^,^ 19. Our results indicate that fibroblasts can be utilized for membrane resealing and blebbing assays to examine whether pharmaceutical candidate compounds restore mutant dysferlin. In addition, diagnosis of dysferlinopathy by immunoblot analysis of skin biopsies is possible.

A previous report revealed that dysferlin is localized in the basal layer of the epidermis, using immunohistochemical analysis 20. In our present study, we showed that dysferlin is expressed in cultured skin fibroblasts from both humans and mice, however, there are no reports of skin abnormalities in dysferlinopathy patients or SJL mice. McNeil *et al.* reported that the percentage of wounded cells in the skin is 3-6%, which is lower than that in skeletal muscle (5-30%) 21. These results may help explain the absence of clinical symptoms in the skin of patients with dysferlinopathy.

We also showed that MG-132 increases the protein levels of truncated or W999C mis-sense mutated dysferlin in fibroblasts. Sinnreich *et al.* showed that proteasome inhibitors restored expression level and biological function of mutant dysferlin in myotubes from a patient carrying the R555W missense mutation, however, there was no significant improvement in the protein expression level of non-sense mutated dysferlin (R1607X) 6 . In accordance with previous report, our results indicated that proteasome inhibitor improved expression level and biological function of mis-sense mutated W999C dysferlin and that proteasomal inhibition was not effective in increasing frame-shift or non-sense mutated dysferlin protein levels. Universal Mutation Database for Dysferlin (UMD-DYSF, v1.4 June 16 2015, http://www.umd.be/DYSF) list 416 disease-causing mutations identified in 843 patients worldwide. UMD-DFSF indicates that approximately 34% of these patients carry mis-sense mutations. Treatment with proteasome inhibitor would be effective a one-third of dysferlinopathy patients.

We previously reported that the transmembrane domain of dysferlin is necessary to increase the protein levels of dysferlin deletion mutants in COS-7 cells 22. Consistent with this report, in the absence of a proteasome inhibitor, the truncated dysferlin protein with a transmembrane domain in SJL/6J mice 10 was expressed at higher levels than truncated human dysferlin (R1607X) lacking the transmembrane domain 6 .

In conclusion, fibroblasts from dysferlinopathy patients and SJL mice showed attenuated membrane repair, and could be a research tool to monitor the effects of drug candidate including proteasome inhibitors on mutant dysferlin.

## Competing Interests

The authors have declared that no competing interests exist.

## Correspondence

The corresponding author can be contacted at cmatsuda@ncnp.go.jp.

## References

[1] Liu J, Aoki M, Illa I, Wu C, Fardeau M, Angelini C, Serrano C, Urtizberea JA, Hentati F, Hamida MB, Bohlega S, Culper EJ, Amato AA, Bossie K, Oeltjen J, Bejaoui K, McKenna-Yasek D, Hosler BA, Schurr E, Arahata K, de Jong PJ, Brown RH Jr. Dysferlin, a novel skeletal muscle gene, is mutated in Miyoshi myopathy and limb girdle muscular dystrophy. Nat Genet. 1998 Sep;20(1):31-6. PubMed PMID:9731526. 9731526 10.1038/1682

[2] Bashir R, Britton S, Strachan T, Keers S, Vafiadaki E, Lako M, Richard I, Marchand S, Bourg N, Argov Z, Sadeh M, Mahjneh I, Marconi G, Passos-Bueno MR, Moreira Ede S, Zatz M, Beckmann JS, Bushby K. A gene related to Caenorhabditis elegans spermatogenesis factor fer-1 is mutated in limb-girdle muscular dystrophy type 2B. Nat Genet. 1998 Sep;20(1):37-42. PubMed PMID:9731527. 9731527 10.1038/1689

[3] Illa I, Serrano-Munuera C, Gallardo E, Lasa A, Rojas-García R, Palmer J, Gallano P, Baiget M, Matsuda C, Brown RH. Distal anterior compartment myopathy: a dysferlin mutation causing a new muscular dystrophy phenotype. Ann Neurol. 2001 Jan;49(1):130-4. PubMed PMID:11198284. 11198284

[4] Bansal D, Miyake K, Vogel SS, Groh S, Chen CC, Williamson R, McNeil PL, Campbell KP. Defective membrane repair in dysferlin-deficient muscular dystrophy. Nature. 2003 May 8;423(6936):168-72. PubMed PMID:12736685. 12736685 10.1038/nature01573

[5] Wang B, Yang Z, Brisson BK, Feng H, Zhang Z, Welch EM, Peltz SW, Barton ER, Brown RH Jr, Sweeney HL. Membrane blebbing as an assessment of functional rescue of dysferlin-deficient human myotubes via nonsense suppression. J Appl Physiol (1985). 2010 Sep;109(3):901-5. PubMed PMID:20558759. 20558759 10.1152/japplphysiol.01366.2009 PMC3774516

[6] Azakir BA, Di Fulvio S, Salomon S, Brockhoff M, Therrien C, Sinnreich M. Modular dispensability of dysferlin C2 domains reveals rational design for mini-dysferlin molecules. J Biol Chem. 2012 Aug 10;287(33):27629-36. PubMed PMID:22736764. 22736764 10.1074/jbc.M112.391722 PMC3431656

[7] Bittner RE, Anderson LV, Burkhardt E, Bashir R, Vafiadaki E, Ivanova S, Raffelsberger T, Maerk I, Höger H, Jung M, Karbasiyan M, Storch M, Lassmann H, Moss JA, Davison K, Harrison R, Bushby KM, Reis A. Dysferlin deletion in SJL mice (SJL-Dysf) defines a natural model for limb girdle muscular dystrophy 2B. Nat Genet. 1999 Oct;23(2):141-2. PubMed PMID:10508505. 10508505 10.1038/13770

[8] Villegas J, McPhaul M (2005) Establishment and culture of human skin fibroblasts. Current protocols in molecular biology / edited by Frederick M Ausubel et al. Chapter 28: Unit 28 23. 10.1002/0471142727.mb2803s71 18265368

[9] Guo LT, Moore SA, Forcales S, Engvall E, Shelton GD. Evaluation of commercial dysferlin antibodies on canine, mouse and human skeletal muscle. Neuromuscul Disord. 2010 Dec;20(12):820-5. PubMed PMID:20817457. 10.1016/j.nmd.2010.07.278 20817457

[10] Vafiadaki E, Reis A, Keers S, Harrison R, Anderson LV, Raffelsberger T, Ivanova S, Hoger H, Bittner RE, Bushby K, Bashir R. Cloning of the mouse dysferlin gene and genomic characterization of the SJL-Dysf mutation. Neuroreport. 2001 Mar 5;12(3):625-9. PubMed PMID:11234777. 11234777 10.1097/00001756-200103050-00039

[11] Philippi S, Bigot A, Marg A, Mouly V, Spuler S, Zacharias U. Dysferlin-deficient immortalized human myoblasts and myotubes as a useful tool to study dysferlinopathy. PLoS Curr. 2012 Feb 2;4:RRN1298. PubMed PMID:22367358. 22367358 10.1371/currents.RRN1298 PMC3274833

[12] Demonbreun AR, Fahrenbach JP, Deveaux K, Earley JU, Pytel P, McNally EM. Impaired muscle growth and response to insulin-like growth factor 1 in dysferlin-mediated muscular dystrophy. Hum Mol Genet. 2011 Feb 15;20(4):779-89. PubMed PMID:21127009. 21127009 10.1093/hmg/ddq522 PMC3024047

[13] Lennon NJ, Kho A, Bacskai BJ, Perlmutter SL, Hyman BT, Brown RH Jr. Dysferlin interacts with annexins A1 and A2 and mediates sarcolemmal wound-healing. J Biol Chem. 2003 Dec 12;278(50):50466-73. PubMed PMID:14506282. 14506282 10.1074/jbc.M307247200

[14] Han R, Campbell KP. Dysferlin and muscle membrane repair. Curr Opin Cell Biol. 2007 Aug;19(4):409-16. PubMed PMID:17662592. 17662592 10.1016/j.ceb.2007.07.001 PMC2144911

[15] Marbini A, Bellanova MF, Ferrari A, Lodesani M, Gemignani F. Immunohistochemical study of merosin-negative congenital muscular dystrophy: laminin alpha 2 deficiency in skin biopsy. Acta Neuropathol. 1997 Aug;94(2):103-8. PubMed PMID:9255383. 9255383 10.1007/s004010050680

[16] Lampe AK, Flanigan KM, Bushby KM, Hicks D. Collagen Type VI-Related Disorders 1993. PubMed PMID:20301676. 20301676

[17] Lampe AK, Flanigan KM, Bushby KM, Hicks D. Collagen Type VI-Related Disorders 1993. PubMed PMID:20301676. 20301676

[18] Dominov JA, Uyan O, Sapp PC, McKenna-Yasek D, Nallamilli BR, Hegde M, Brown RH Jr. A novel dysferlin mutant pseudoexon bypassed with antisense oligonucleotides. Ann Clin Transl Neurol. 2014 Sep;1(9):703-20. PubMed PMID:25493284. 25493284 10.1002/acn3.96 PMC4241797

[19] Wein N, Avril A, Bartoli M, Beley C, Chaouch S, Laforêt P, Behin A, Butler-Browne G, Mouly V, Krahn M, Garcia L, Lévy N. Efficient bypass of mutations in dysferlin deficient patient cells by antisense-induced exon skipping. Hum Mutat. 2010 Feb;31(2):136-42. PubMed PMID:19953532. 19953532 10.1002/humu.21160

[20] Vainzof M, Anderson LV, McNally EM, Davis DB, Faulkner G, Valle G, Moreira ES, Pavanello RC, Passos-Bueno MR, Zatz M. Dysferlin protein analysis in limb-girdle muscular dystrophies. J Mol Neurosci. 2001 Aug;17(1):71-80. PubMed PMID:11665864. 11665864 10.1385/JMN:17:1:71

[21] McNeil PL, Steinhardt RA. Loss, restoration, and maintenance of plasma membrane integrity. J Cell Biol. 1997 Apr 7;137(1):1-4. PubMed PMID:9105031. 9105031 10.1083/jcb.137.1.1 PMC2139853

[22] Matsuda C, Kameyama K, Tagawa K, Ogawa M, Suzuki A, Yamaji S, Okamoto H, Nishino I, Hayashi YK. Dysferlin interacts with affixin (beta-parvin) at the sarcolemma. J Neuropathol Exp Neurol. 2005 Apr;64(4):334-40. PubMed PMID:15835269. 15835269 10.1093/jnen/64.4.334

